# Validation of miRNA prognostic significance in stage II colorectal cancer: A protocol for systematic review and meta-analysis of observational clinical studies

**DOI:** 10.1097/MD.0000000000014570

**Published:** 2019-03-22

**Authors:** Shanthi Sabarimurugan, Chellan Kumarasamy, Madhav Madurantakam Royam, Karthik Lakhotiya, Gothandam Kodiveri Muthukaliannan, Suja Ramalingam, Rama Jayaraj

**Affiliations:** a School of Biosciences and Technology, Vellore Institute of Technology (VIT), Vellore, Tamil Nadu, India; b University of Adelaide, North Terrace Campus, Adelaide South Australia 5005, Australia; c Department of Biochemistry, Bharathiyar University, Coimbatore, Tamil Nadu, India; d College of Health and Human Sciences, Charles Darwin University, Ellengowan Drive, Darwin, Australia.

**Keywords:** Colorectal cancer, data analysis, meta-analysis, miRNA, PRISMA, prognosis, publication bias, stage II, systematic review

## Abstract

**Background:**

Advances in early detection and treatment of colorectal cancer (CRC) has seen marked improvements in recent years. However, it is still the third leading cause of cancer-related deaths worldwide. Although there are studies reporting the significance of miRNAs in stage II colorectal cancer, there is no known comprehensive study utilising collective data from multiple published studies. Thus, this study will focus on performing a systematic review and meta-analysis using published data to identify and underline multiple miRNA targets, with regard to disease prognosis in stage II CRC patients.

**Methods:**

The systematic review will be reported according to the Preferred Reporting Items for Systematic Review and Meta-Analysis (PRISMA) guidelines, 2015 statement. The required articles will be obtained via a search of bibliographical databases such as Cochrane Library, EMBASE, MEDLINE, PubMed, and Web of Science. A set of keywords in multiple permutations will be used for the search. The reference lists of included studies will also be manually searched to further increase the robustness of the search results. The systematic review will primarily be done via a literary synthesis.

**Results:**

Quantitative data synthesis will be based on estimated effect across the studies with forest plots generated to observe pooled outcome measures. A fixed or random-effect model of meta-analysis will be used depending upon the heterogeneity observed between studies. Subgroup analysis will be carried out depending on the availability of sufficient clinical data.

**Conclusion:**

Based on the systematic review and meta-analysis results of this study, the possible miRNAs could be predicted toward the prognosis of stage II colorectal cancer, and specific miRNAs may perhaps be considered as a biomarker and therapeutic target for this malignancy.

## Abstract 2

Supplemental Digital Content is available in the text

## 1 Introduction

### 1.1 Background

Colorectal cancer (CRC), also known as colon cancer or bowel cancer, is cancer originating from colon or rectal lining, where healthy cells transform into cancerous cells, leading to the formation of large, rapidly growing tumors through the blood and lymphatic system which remain benign or become malignant as the disease progresses.^[1]^ Among all the four stages of CRC, stage II needs prior attention due to the existence of recurrence in CRC patients and the effectiveness of the adjuvant chemotherapy has not produced better result till date when compared to all other stages.^[2]^ In general, CRC has been classified as five stages which are: stage 0 to IV. Stage 0 to stage I has been categorised as Submucosal invasion, Stage II is classified as penetration of the outer colonic wall, Stage III is classified as Lymph node invasion and finally stage IV as metastasis.^[3]^ Patients with stage I colon cancer determines that the tumors are not invaded through the colonic wall, and it may likely fall into the first group. These patients do not receive any kind of Adjuvant chemotherapy. Stage III colon cancers are as it spreads to local lymph nodes, which are likely fall into second and third groups for which chemotherapy is provided by multiple large clinical trials. It is to be noted that stage II colon cancer will be comprised as a heterogeneous combination and would likely fall into all the three groups which are the importance of stage II riskness and hence it should be studied more in detail with a proved meta-analysis examination.^[4]^

### 1.2 Epidemiology

CRC is the third most commonly diagnosed malignancy and the fourth leading cause of cancer death in the world, accounting for about 1.4 million new cases and almost 700,000 deaths in 2012.^[5]^ The Australian government's (Cancer Australia) 2017 estimate suggests 16,682 (12.4%) new cases out of which 4114 (8.6%) cases were reported as leading to death.^[6]^ A report from American Cancer Society declared that reliable statistics on deaths from colon and rectal cancers separately are not available due to almost 40% of deaths from rectal cancer being misclassified as colon cancer on death certificates.^[7]^ Although surgical resection can be highly useful for localised disease, 25%–40% of patients develop recurrence after surgery in stage II CRC patients.^[8]^ The recurrence of CRC is, for the most part, a time-limited phenomenon, with 40%–50% of the recurrences becoming apparent within the first year after the initial surgical resection.^[8]^

### 1.3 Rationale

#### 1.3.1 The importance of the issue

The challenge of the high mortality associated with CRC is that it is asymptomatic in the initial stages after treatment, where there are limited methods of monitoring patient's clinical outcomes after chemotherapy for early-stage CRC. Colonoscopy screening has contributed to the early recognition of CRC and subsequent reduction in mortality in recent years.^[9]^ Furthermore, there is a room for improvement in the treatment for recurrent and metastatic CRC. Screening with carcinoembryonic antigen (CEA) levels in the blood has also been shown to have poor sensitivity (36%–74%, based on the stage of CRC).^[10]^ Therefore, the identification and confirmation of noninvasive or minimally invasive circulating biomarkers for CRC disease progress and remission, and cancer prognosis remains a quandary, prompting an investigation in CRC biomarker research.

There is a need for detailed analyses regarding prognostic biomarkers, specifically in stage II CRC. Surgical resection is a primary treatment modality in stage II and stages III CRC where the patients show poor survival and develop recurrent disease. The underlying cause behind the poor prognosis is not well defined, prompting further investigation.^[11]^ Furthermore, when compared to stage III CRC, stage II CRC suffers more uncertainty regarding treatment methods has proven to be effective in stage III CRC. It is seen that stage III CRC patients benefit from adjuvant chemotherapy. However, its effects on stage II CRC is still under deliberation.^[12]^ An improvement in disease-free survival (DFS) was observed in stage III CRC patients, after the addition of oxaliplatin to the treatment strategy, while its efficacy in stage II CRC remains unproven.^[13]^ Patients with stage II disease are a heterogeneous population and the subgroups, and different tumor location may help the clinicians to determine the appropriate treatment course.^[14]^ For example, stage II patients with T4 primary tumors have a 72% 5-year overall survival rate, which is worse than that of patients with a T2 primary tumor with involvement of fewer than four lymph nodes (83% 5-year overall survival).^[14]^ A comprehensive systematic review and meta-analysis consisting of data accumulated from a broad set of published studies will allow us to explore the implication of prognostic specific miRNA expression in CRC patient survival. Hence, the proposed study will focus on the investigation of the overall effect of miRNA expression on stage II CRC patient's survival.

#### 1.3.2 Association of miRNA expressions and CRC patients prognosis

Several studies have analysed miRNA expressions in CRC and interpreted their significance in clinical prognosis and survival.^[15–17]^ It is now well recognised that the aberrant expression of miRNAs is connected with cancer development, progression and treatment.^[18,19]^ Some studies have also identified some miRNAs as potential diagnostic and predictive biomarkers.^[20]^ Some miRNAs were also reported to be either over-expressed, under-expressed or dysregulated in CRC.^[21,22]^ However, despite significant methodological progress, concrete biomarkers capable of guiding treatment, have not yet been identified.

Although few meta-analyses have been performed on miRNA expressions and CRC patients prognosis (in all stages of CRC) the findings were inconclusive and inconsistent on prognostic specific miRNAs in stage II CRC patients’.^[23–26]^ Unfortunately, they did not produce the focussed meta-analysis on CRC stage II patients alone. Our proposed study will study and interpret the results which would be more recent (2012–2018) and focus on stage II CRC patients alone.

#### 1.3.3 How will the study address this issue?

The primary treatment modality of surgery for stage II CRC, not only suffers from a 25% recurrence rate but also from the patients exhibiting poor survival after primary surgery who are not surviving beyond the 5-year mark.^[27]^ This gap in existing knowledge is only exacerbated by the scarcity of clinical data pertaining specifically to stage II CRC. Therefore, there is an urgent need to explore and establish prognostic biomarkers, such as prognostic specific miRNAs, which may allow for better clinical treatment strategies for stage II CRC patients. This protocol aims to provide strategies for a systematic review and meta-analysis of prognostic specific miRNAs in stage II CRC.

#### 1.3.4 How will it help?

The proposed meta-analysis and systematic review of the expression of various miRNAs and their implications for the survival of CRC patients will be performed based on previously published studies. This will allow us to identify the possible biomarkers for prognosis, and monitoring the CRC patients in the post-treatment state. This study will be constructed with various parameters in mind, including miRNA expression, tumor anatomic location, ethnic variations, and risk factors involved in CRC. Therefore, a systematic review and meta-analysis on prognostic specific miRNA expression in stage II CRC is a crucial step in highlighting patient clinical outcome patterns which will inform clinical decision makers on CRC treatment strategies.

## 2 Methods and analysis

This study aims to evaluate the prognostic effect of miRNA expression in stage II CRC patients. The research protocol follows the PRISMA-P (Preferred Reporting Items for Systematic Reviews and Meta-Analysis Protocol), 2015 guidelines for systematic review and meta-analysis.^[28]^

**PROSPERO registration number**: The study was registered in PROSPERO and was assigned the registration ID: CRD42017080631.

### 2.1 Search strategy

**Figure 1:**
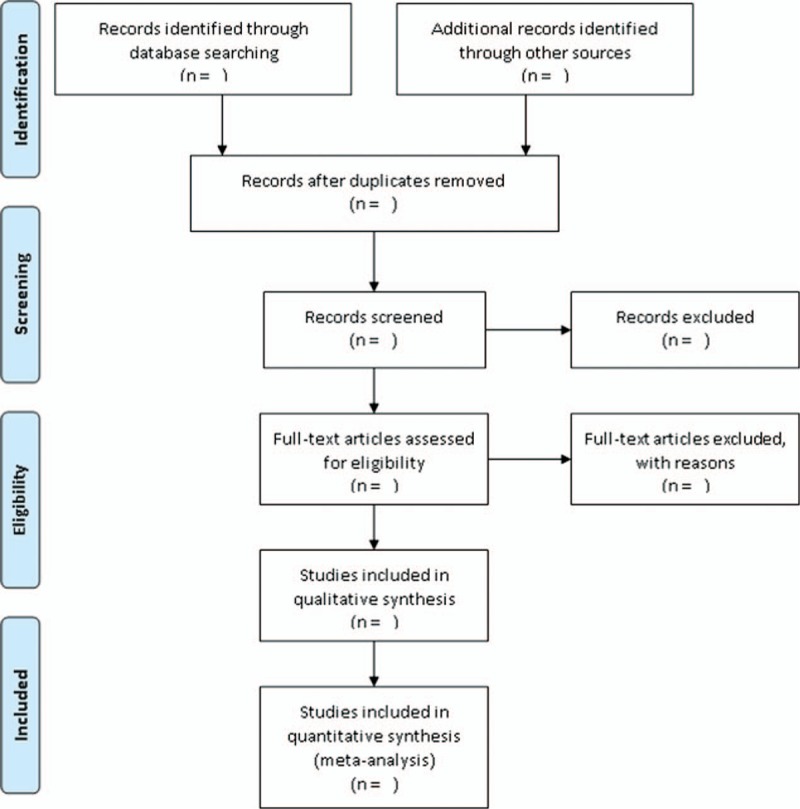
Schematic representation of the selected articles.

A comprehensive search strategy will be developed to obtain suitable peer-reviewed literature. A three steps search strategy will be utilized in this systematic review. An initial limited search of online bibliographic databases such as Cochrane library, EMBASE, PubMed, Science Direct, SCOPUS and Web of Science will be undertaken followed by analysis of the words contained in the title and abstract and of the index terms will be used to describe each relevant article. A full search strategy for PubMed as an example is detailed in Table 1. The second step will be considered by using all the keywords and index terms across all the included studies. The final step will be extracted using the reference list from the identified reports and articles for collecting additional studies. Published research may also be used as viable data sources if a novel or critical findings in regard to CRC prognosis and miRNA expression will be reported. The complete search strategy will be depicted in a flow diagram (Fig. 1).

**Table 1:**
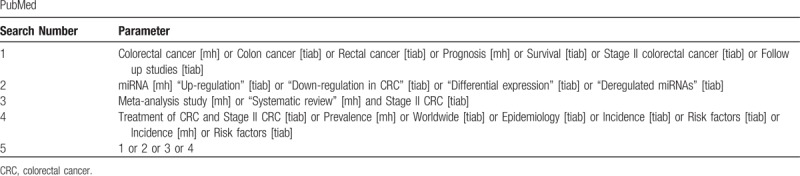
The initial search strategy.

### 2.2 Study selection process

The primary screening will be based on the relevance of the paper to the topic of the study. The studies enrolled will be extracted from several bibliographic databases between 2012 and 2018 using multiple keywords according to the PRISMA guidelines. The screening will be performed individually by two authors blinded to each other's results of selection, upon a cursory examination of titles and abstracts of selected articles. All articles deemed to be suitable by either author will be included for further selection. The final selection will be based on the defined inclusion and exclusion criteria. The discrepancies that arise will be dealt with by deliberation and debate between the two authors. A third reviewer will be involved during this process to act as the final tie-breaker vote in case of the discussion arriving at a standstill. Manual checking will be done from the back references and review article in conscious to avoid missing any appropriate articles. All reviewers will record a specific reason for excluding studies, along with an explanatory note for their determination. A diagrammatic representation detailing the selection process will be a part of the final systematic review and meta-analysis.

### 2.3 Selection criteria

The studies fulfilling all the following criteria will be included in the systematic review.

Inclusion criteria

- Studies that discuss the clinicopathological characteristics of stage II CRC patients along with hazard ratio will be included.
- Studies that discuss a tumor, lymph node, and metastasis (TNM) stage of CRC patients will be included.
- Studies that published the miRNA deregulation in stage II CRC patients will be included.
- Articles that discuss the survival outcomes of stage II CRC patients will be included for meta-analysis.
- Studies published only with the reasonable sample size (above 30) will be included for systematic review.
- Studies providing Kaplan Meier curve will also be included for systematic review even though if the concern studies failed to provide HR values.

Exclusion criteria

- Duplicated data will be rejected, If the same data has been used in multiple publications
- Data sources consisting of primarily subjective analysis, with no primary data or method of analysis will be excluded.
- Studies excluded if it is not published in English.
- Excluded if there is no full text or any conference abstracts.
- Letters to the editors will be excluded.

### 2.4 Data extraction and management

Data will be extracted from the studies included in the systematic review and meta-analysis by two independent reviewers. A standardized, Microsoft Excel master sheet framework will be developed to record data extracted from each qualified study for evaluation of study quality and data synthesis. Full texts of the articles will be analysed, along with all available figures and tables to obtain pertinent CRC data for the systematic review and meta-analysis.

### 2.5 Types of studies

This review will consider the observational studies (including cohort studies, cohort studies, case–control studies, cross-sectional studies, and retrospective experimental studies) which discuss the miRNA prognosis in stage II CRC patients. The case reports will be excluded from the systematic review where only published data from the full-text articles will be included in the meta-analysis. Studies which report Hazard Risk and Confidence Intervals on patients survival will be considered. Also, studies, which demonstrate other clinicopathological parameters, will be considered for subgroup analysis. Only studies published in English will be considered for inclusion. The following studies will be excluded such as multiple case reports, comparative study without case controls, prognosis results without evaluating miRNA dysregulation, treatment preferences with miRNA dysregulation results, prevalence, and epidemiological analysis in CRC patients.

### 2.6 Participants

This proposed study will involve clinical data of the patient cohorts from the included studies that have been identified through the online search. There will not be any limitations of gender, age, lymph nodes, or any treatment parameters. The platform for the diagnosis of disease from patients will be noted with the results. The mode of treatment will be recorded with the results of pre- and post-outcome measurements. The patient samples and source of the miRNA identification will be observed from individual study and investigate the expression of miRNAs in stage II CRC patients. The follow-up period will be extracted from the included studies.

### 2.7 Data items

The extracted data elements of this review will include the following.

1. Author name and publication year, Place/Country patients enrolled in each study, size of CRC patient cohort.
2. Demographic characteristics (including author, year of publication, a geographic region, the study period, age, gender, study design, follow up period, type of research, sample size and sampling procedures, the validity of confirmative diagnosis, and method of data collection), (including International Classification of Disease (ICD) Code for the anatomical site of cancer under study, number of CRC cases/patients).^[29]^
3. Clinicopathological characteristics of study participants such as Tumor location (Right/left colon, rectum or stroma), TNM (tumor, node, and metastases) staging (T_2_-T_4_/N_0_/M_0_), histological type (non-mucinous/mucinous), neuronal invasion (positive/negative), lymphovascular invasions (positive/negative), and microsatellite malignancy grade (high/low).^[30]^
4. Biological characterization of stage II CRC will also be recorded. Depending upon the patient history, the biological incidence could be observed whether it is sporadic form, family type, or hereditary factor occurs.^[31]^ Because there is a chance of established stages ranging from adenomatous lesions to the manifestation of a malignant tumor will occur from inheritance patterns and family predisposition.^[31]^
5. This helps to find out the impact of miRNA on patients survival. Adjuvant therapy details if available, carcinoembryonic antigen (CEA) level determination, microsatellite instability (MSI) testing (positive/negative), E-cadherin (low/high), fecal occult blood test, metastasis-associated protein (MTA-1protein) (low/high) and other invasive noninvasive CRC confirmation will be noted if the information is provided with appropriate results.^[32]^
6. The methodology used for the miRNA quantification along with the source of the samples, miRNA expression in colon and rectal cancers separately, upregulated, downregulated, and deregulated miRNAs, miRNA expression rates during the follow-up period, HR estimates with 95% confidence interval (CI) for overall survival (OS), disease free survival (DFS) and disease-specific survival (DSS).

### 2.8 Data synthesis and management

Data synthesis will be of two forms. The literary analysis of the data will be done in the form of a systematic review. The review will focus upon each study, analysing the results and the variables contributing to the variations found in each study. An analytical approach will be taken to resolve any discrepancies observed in the studies when compared to the overall pattern of survival and related miRNA expression rates observed in the published studies. All exceptions observed will be analysed and justifications will be given, either based on within-study information or from supporting data obtained from past publications. Citation software manager, endnote will be used for reference and selection process.^[33]^

### 2.9 Assessment of methodological quality

The methodological quality will be assessed by the quality assessment template based on the National Heart, Lung and Blood Institute (NHLBI) for observational cohort and cross-sectional studies.^[34]^ The Assessment was followed from previously published protocol and studies.^[35,36]^ This assessment template will be used to evaluate the selected full-text studies which will be considered eligible for systematic review (rated as good, fair, and poor). There are 14 elements to be analysed and to be rated which are tabulated in Table 2. They include sample size, population, follow up period, survival outcome, the assessment will be based on the specific study requirements for systematic review and meta-analysis and will be subjectively analysed by the reviewers. All disagreements will be resolved by a vote from a third, neutral reviewer.

**Table 2:**
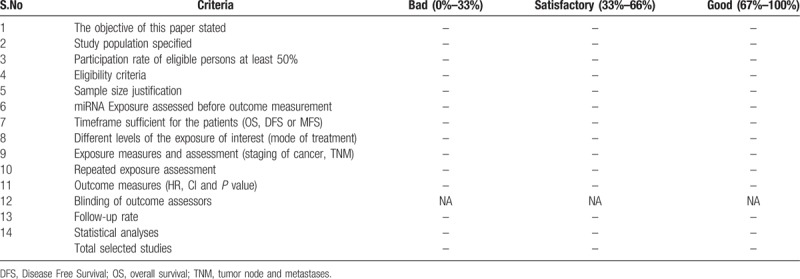
Quality assessment of the selected studies.

### 2.10 Publication bias

To any systematic review and meta-analysis study, publication bias will be considered as basic priority assessment which is mandatory in regard.^[37]^ Publication bias will be assessed visually by the symmetry of funnel plots constructed with HR and 95% CI values for CRC patient survival. The quantitative analysis of publication bias performed by utilizing the Funnel plot, ‘Orwin's and classic fail-safe N test’,^[38]^ ‘Begg and Mazumdar Rank correlation test,^[39]^ Harbord-Egger's Test of the intercept ^[40]^ and ‘Duval and Tweedie's trim and fill’ calculation.^[41]^

### 2.11 Statistical analysis

#### 2.11.1 Assessment of heterogeneity

Meta-analysis, a well-established method of analysing prognostic effects and differences using information from multiple clinical and observational studies to obtain precise effect size estimates, will be used as the quantitative method of data synthesis in this study. Comprehensive meta-analysis (CMA) software (version 3.3.070) will be used to perform the meta-analysis to generate forest plots using HR and associated 95% CIs of overall survival, disease-free survival and disease-specific survival or various endpoints obtained from the selected studies. The meta-analysis will be based on the application of a random effects model or fixed effects model, based on between-study heterogeneity. The mean effect of HR will be used to analyse the possibility of clinical utility from the presented results.^[42]^ Heterogeneity will be based on Higgin's *I*^2^ statistic and Cochran's *Q*-test. The threshold values to determine heterogeneity will be based upon the studies selected with 25%, 50%, and 75% tentatively indicating a low, moderate and high heterogeneity respectively.^[43]^ Cochran's *Q* test will be used as a secondary heterogeneity detection tool due to its low power of heterogeneity detection. In case of any discrepancy, the *I*^2^ statistic will be given precedence.^[44]^ The substantial heterogeneity *I*^2^ statistic would be indicated the value more than 50%.^[45]^ Both *I*^2^ statistic and *Q* test ignore threshold effect,^[46]^ and hence tau squared test will be assessed to estimate the variation between the effects of test accuracy observed in different studies.^[47,48]^ The forest plots generated will be interpreted and analysed to elucidate the outcome effects and effect estimates of different miRNA in determining patient survival in CRC. Meta-analysis will be done if sufficient data is available and found.

#### 2.11.2 Subgroup analysis and meta-regression

Subgroup analysis or meta-regression will be considered based on the characteristics which may offer a better resolution into the outcome effects observed in primary meta-analysis.^[49]^ The most predictable tentative groups of subgroup analysis were age, gender, miRNA, and risk factors. Subgroup analysis will help to produce high resolution from the total meta-analysis, which influences the overall survival outcome effect.^[50]^ Based on the clinico-pathological and biological factors along with the available data from tumor location, tumor grade, the risk of recurrence, adjuvant therapy will also be considered as additional parameters for subgroup analysis. The meta-regression analysis will be conducted as an additional parameter which is based on heterogeneity relative contribution on one or more key variables on various endpoints by regression analysis technique.^[51]^

## 3 Discussion

Despite a comprehensive understanding of treatment after surgical resection in the CRC, the treatment for stage II colorectal cancer still offers room for development. The association between miRNA expression rates and patient age, sex, as well as clinicopathological parameters, such as tumor size, differentiation, location, invasion depth, metastasis, TNM stage, patient survival, and ethnic variations, contributes to the outcome effects observed in each study. These factors must also be analysed to identify and underline the significant miRNA influencing the outcome effects of survival observed in the cumulative data. The current 5-year survival rate for stage II colorectal cancer patients is between 70% and 80%. Surgery has remained the standard treatment option for stage II colorectal cancer. However, about 30% of stage II patients will relapse, and there is no reliable biomarker to determine which patients are at high risk and should be managed with adjuvant chemotherapy.^[52]^ As for advanced stage III and IV colorectal cancer patients, despite years of effort, there is still a lack of highly reliable prognostic biomarkers to determine which patients will benefit from chemotherapy. In both early and advanced stages of colorectal cancers, there is an unmet need for biomarkers for better clinical management. A need that verified miRNAs as biomarkers may be capable of fulfilling.

Several studies have interpreted the significance of miRNA expression in stage II colorectal cancer, where the outcome (survival) depended on the over-expression or under-expression of a particular miRNA expression signal. On observing the importance of miRNA in numerous articles, we plan to verify and validate specific miRNA as a novel biomarkers for the monitoring and prognosis of stage II CRC. We are thereby allowing for new, patient-friendly, effective approaches to the prognosis of stage II CRC patients, and providing clinicians better assessment tools for CRC patients. Thus, opening the door to a possibility of new treatment options concerning stage II CRC.

## 4 Assessment of protocol summary

The study protocol was followed as per the guidelines from PRISMA P guidelines,^[28]^ and the PRISMA checklist^[53]^ will be provided as Supplementary Table 1. The selection process will be reported as a flow diagram (Fig. 1) as specified in the PRISMA guidelines.^[54]^ This will include the list of excluded studies and the reasons for exclusion. In-text descriptions will be used to describe the qualitative data in the studies. We will present the output of our meta-analysis data in forest and funnel plots. The quantitative data will include literary reports of the figures, charts, and diagrams.

## 5 Strengths and limitations of this study

- The protocol follows the PRISMA-P guidelines.
- This is the first study of its kind, with a comprehensive analysis of the prognostic effects of miRNA in stage II CRC in the form of a systematic review and meta-analysis.
- Since the study focuses on the systematic review of stage II colorectal cancer, the number of articles may be lower when compared to other colorectal studies.
- Reporting bias may occur due to the exclusion of articles published in languages other than English, articles published before 2009 and unpublished articles. This is a necessary step to ensure that the results of this study are specific, currently relevant and accurately replicable.

## Acknowledgment

We would like to acknowledge the Meta-analysis concepts and applications workshop manual by Michael Borenstein for his guidelines on reporting Meta-analysis, subgroup analysis and publication bias (www.meta-analysis-workshops.com).

## Author contributions

RJ conceived this study and provided supervision and mentorship. SS led the development of the study protocol and design, wrote the first draft of the protocol, and coordinated and integrated comments from co-authors RJ, CK, MRM, KL, GKM and RS critically revised and edited successive drafts of the manuscript and gave input to the final draft of the protocol. RJ provided methodological guidance on the overall development of the protocol. All the authors read, refined and approved the final version of the manuscript.

**Conceptualization:** Rama Jayaraj.

**Formal analysis:** Rama Jayaraj.

**Investigation:** Shanthi Sabarimurugan, Rama Jayaraj.

**Methodology:** Shanthi Sabarimurugan, Rama Jayaraj.

**Project administration:** Shanthi Sabarimurugan, Chellan Kumarasamy, Madurantakam Royam Madhav, Rama Jayaraj.

**Resources:** Rama Jayaraj.

**Software:** Karthik Lakhotiya.

**Supervision:** KM. Gothandam, Suja Ramalingam, Rama Jayaraj.

**Validation:** Suja Ramalingam, Rama Jayaraj.

**Writing – original draft:** Shanthi Sabarimurugan, Chellan Kumarasamy, Madurantakam Royam Madhav, Karthik Lakhotiya, KM. Gothandam, Suja Ramalingam, Rama Jayaraj.

**Writing – review & editing:** Shanthi Sabarimurugan, Chellan Kumarasamy, Madurantakam Royam Madhav, Karthik Lakhotiya, KM. Gothandam, Suja Ramalingam, Rama Jayaraj.

Rama Jayaraj orcid: 0000-0002-2179-0510.

## Supplementary Material

Supplemental Digital Content
